# New Sights Into Long Non-Coding RNA LINC01133 in Cancer

**DOI:** 10.3389/fonc.2022.908162

**Published:** 2022-06-07

**Authors:** Shengnan Jiang, Qian Zhang, Jiaqi Li, Khadija Raziq, Xinyu Kang, Shiyin Liang, Chaoyue Sun, Xiao Liang, Di Zhao, Songbin Fu, Mengdi Cai

**Affiliations:** ^1^ Key Laboratory of Preservation of Human Genetic Resources and DiseaseControl, Ministry of Education, Harbin Medical University, Harbin, China; ^2^ Laboratory of Medical Genetics, Harbin Medical University, Harbin, China; ^3^ Department of Genecology and Obstetrics, The Second Affiliated Hospital of Harbin Medical University, Harbin, China

**Keywords:** LINC01133, cancer, CeRNA, RBP, TME, transcription, LncRNA

## Abstract

LINC01133 is a long intergenic non-coding RNA that regulates malignancy in several cancers, including those of the digestive, female reproductive, respiratory, and urinary system. LINC01133 is an extensively studied lncRNA that is highly conserved, and its relatively stable expression is essential for its robust biological function. Its expression is highly tissue-specific with a distinct subcellular localization. It functions as an oncogene or a tumor suppressor gene in different cancers *via* multiple mechanisms, such as those that involve competing with endogenous RNA and binding to RNA-binding proteins or DNA. Moreover, the secretion and transportation of LINC01133 by extracellular vesicles in the tumor micro-environment is regulated by other cells in the tumor micro-environment. To date, two mechanisms, an increase in copy number and regulation of transcription elements, have been found to regulate LINC01133 expression. Clinically, LINC01133 is an ideal marker for cancer prognosis and a potential therapeutic target in cancer treatment regimes. In this review, we aimed to summarize the aforementioned information as well as posit future directions for LINC01133 research.

## Introduction

Long noncoding RNAs (lncRNAs) are vital regulators of cancer progression. Several studies have reported various mechanisms of regulation of cancers by lncRNA. However, not all lncRNAs are functionally significant in cancer; and their function is highly conserved and tissue specific (1, 2). Recently, a set of star lncRNAs with significant functions had been identified. Of these, LINC01133 has been found to be a regulator of cancers of the digestive, female reproductive, urinary, respiratory, and skeletal systems. The carcinogenic nature of high LINC01133 expression levels has been reported in renal cancer (3), cervical cancer (4),Lung adenocarcinoma (5) and sarcoma (6). However, LINC01133 also acts as a tumor suppressor that inhibits the invasion and metastasis of cancer cells in bladder cancer (7), nasopharyngeal carcinoma (8), oral epithelial cancer (9)and melanoma cancer (10).

As per the National Center for Biotechnology Information database (https://www.ncbi.nlm.nih.gov), LINC01133 is located on the long arm of chromosome 1, zone 2 (1q32.2; hg38 159,961,224-159,979,086 bp) and has four transcripts: ENST00000635112.1, ENST00000657602.1, ENST00000423943.2, and ENST00000443364.6. The transcript ENST00000635112.1 has 1996 bases and 3 exons, the transcript ENST00000657602.1 has 1418 bases and 4 exons, the transcript ENST00000423943.2 has 1405 bases and 3 exons, and the transcript ENST00000443364.6 has 1266 bases and 4 exons. LINC01133 is a long intergenic non-coding RNA (lincRNA) that does not share sequences with the coding regions of the genes present on chromosome 1. LincRNAs bind to DNA (11), RNA (6), and proteins (6) and mediate various functions; such as chromatin and genome architecture remodeling, RNA stabilization, and enhancer-associated transcription (12–14).

The subcellular localization of lncRNAs is closely correlated to their function. LINC01133 is located in both the nucleus and cytoplasm, and this subcellular localization depends on the type of cancer. For example, LINC01133 tends to localize in both the nucleus and cytoplasm in ovarian cancer (15), and it tends to localize in the cytoplasm rather than in the nucleus in gastric cancers (16).

Based on its genomic location (intergenic), structural features, and subcellular localization, LINC01133 regulates biological processes in cancers *via* multiple mechanisms that are activated simultaneously. For example, LINC01133 exerts tumor suppressor effects in hepatocellular carcinoma not only through the competing endogenous RNAs (ceRNA) mechanism that involves binding to miR-199a-5p, but also by binding to the ANXA2 protein (17). In this review, we describe the molecular mechanism underlying the various biological functions of LINC01133, its role in cancer, and future research directions.

## Subcellular Localization and Function of LINC01133

**Figure 1:**
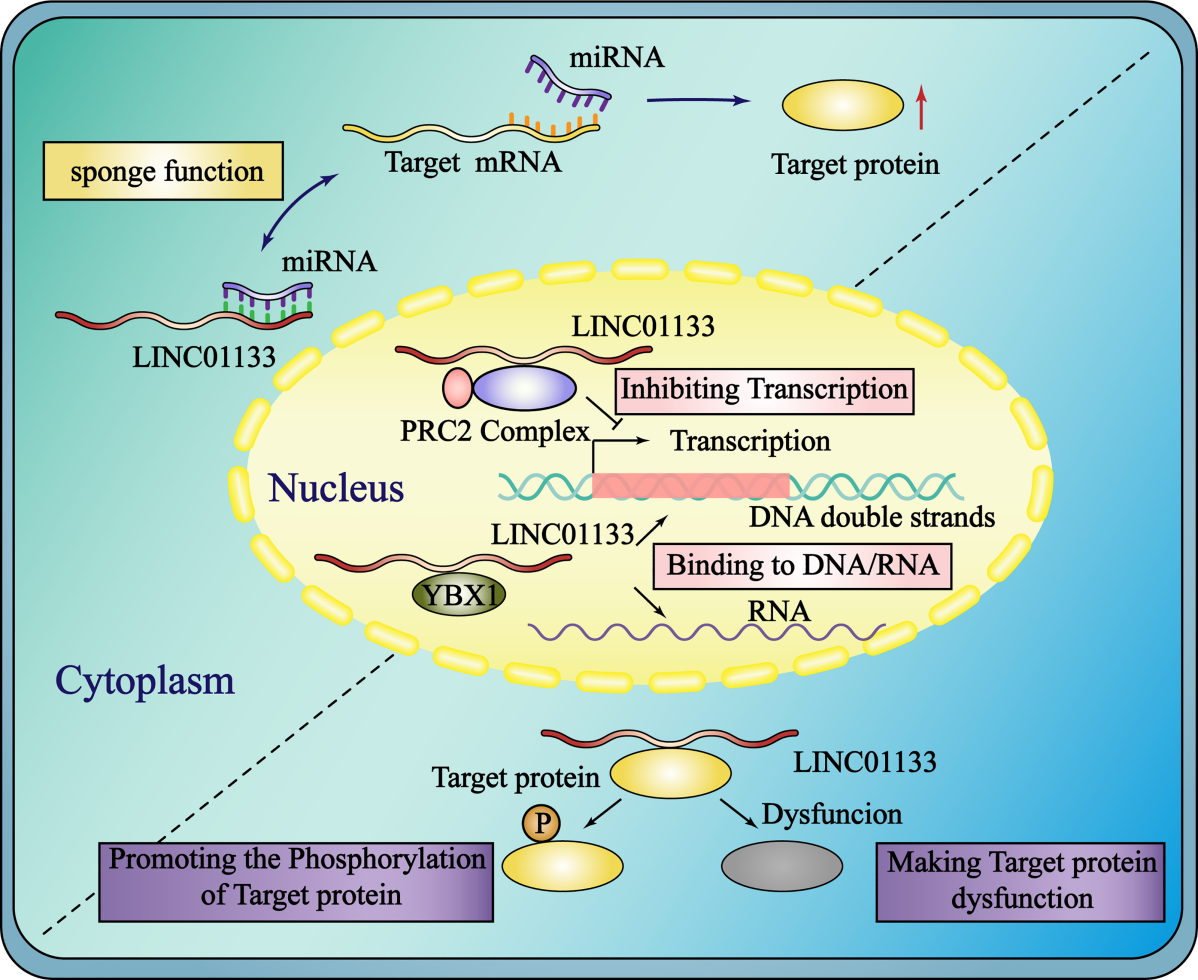
Subcellular localization and function of LINC01133. In the cytoplasm, LINC01133 functions by the mechanisms of ceRNA and binding to RBPs which promotes the phosphorylation of target protein or makes it dysfunction. In the nucleus, LINC01133 functions by the mechanisms of inhibiting transcription and binding to DNA or RNA. The blue rectangle represents the cytoplasm and the yellow oval represents the nucleus. The black dashed line separates the different mechanisms of LINC01133 in the cytoplasm.

The subcellular localization of lncRNA largely determines its biological function. Post-transcriptional regulation is mainly mediated by the ceRNA mechanism, whereas translation and post-translational protein degradation are mediated by binding to the protein in the cytoplasm (18). Linc00483 has been found to act as a potential tumor suppressor in colorectal cancer (CRC) *via* ceRNA mechanism in the cytoplasm (19). Cytoplasmic lncRNA ALAL-1 regulates lung cancer immune evasion by binding to SART3 and affecting nuclear translocation of USP4 in (20).One of the most important mechanisms which ncRNA regulates cancer progression is interacting with miRNA or protein. CircRNA, another famous ncRNA, binds to miRNA or protein to exert the function in cancer. CircSMARCA5 acts as a tumor suppressor by binding SRSF1 in glioblastoma (21)or binding miR-181b-5p/miR-17-3p-TIMP3 axis in prostate cancer (22). When lncRNAs are localized in the nucleus, they regulate gene transcription or pre-transcription levels by binding to the DNA promoter region or transcription factors, respectively. LncRNA Haunt competitively interacts with enhancer-promoter region of *HOXA* gene during embryonic stem cell differentiation (23). The interaction between lncRNA CCAT1-Land transcription factor CTCF has a vital role in mediating *MYC* chromatin looping in CRC (24). The primary and most prevalent way for LINC01133 to the regulate biological progression of cancers is through the ceRNA and protein binding mechanisms in the cytoplasm and the regulation of transcription in the nucleus (**Figure 1**).

### LINC01133 Regulates miRNA and mRNA Levels in the Cytoplasm *via* the ceRNA Mechanism

**Figure 2:**
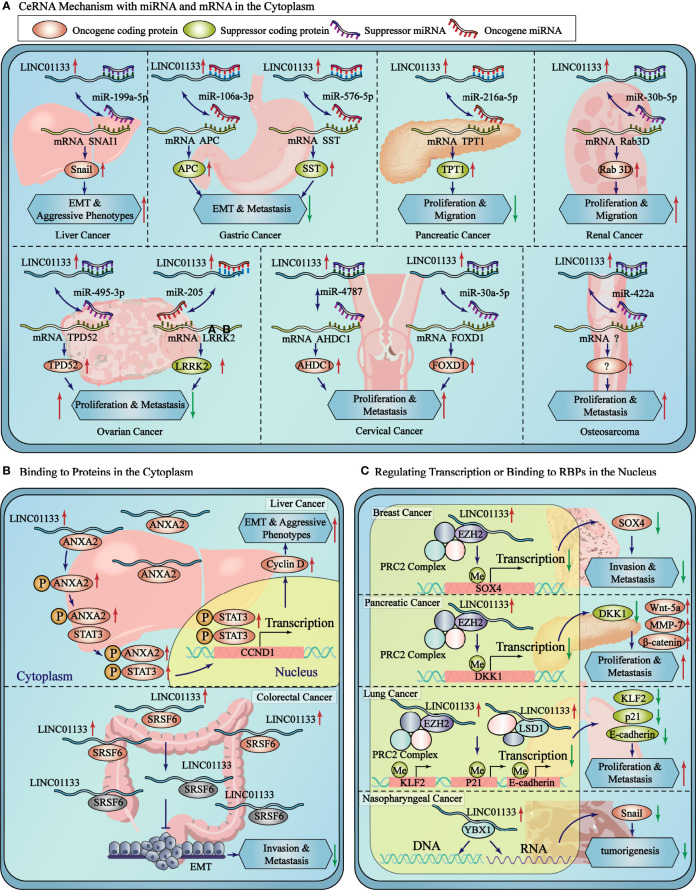
LINC01133 is involved in ceRNA regulation and protein binding in cytoplasm and protein/DNA binding in nucleus. **(A)** The detailed ceRNA mechanisms of LINC01133 in the cancers of different tissue type. **(B)** LINC01133 bounds to proteins in cytoplasm to regulate the modification or activity of the target proteins. **(C)** LINC01133 bounds to proteins or DNA in nucleus to regulate the transcription of target genes. The blue rectangle represents the cytoplasm, and the yellow block represents the nucleus. The black dashed lines separate the cancers of different tissue types.

ceRNAs are transcripts that competitively bind to miRNAs to regulate their abundance. In a ceRNA network of lncRNA-miRNA-mRNA, the miRNA directs the RNA-induced silencing complex by binding to the miRNA response element to inhibit protein production by inhibiting translation or promoting mRNA degradation. However, lncRNAs compete with mRNA to bind to miRNAs and attenuate the inhibition of mRNA translation to restore expression levels (25) (**Figure 2A**).

The ceRNA mechanism of regulation by LINC01133 is commonly observed during the regulation of malignancy of cancers. Studies have reported that LINC01133, which is mainly enriched in the cytoplasm of gastric cancer cells, binds to miR-106a-3p and promotes the expression of proteins of the anaphase promoting complex (APC). Overexpression of the APC blocks the nuclear accumulation of β-catenin, leading to the inactivation of Wnt signaling; in turn inhibiting the proliferation, migration, and epithelial-mesenchymal transition (EMT) of gastric cancer cells (26). LINC01133 was also found to be enriched in the cytoplasm of liver cancer cells. It sponges up miR-199a-5p, leading to an increase in the expression of SNAI1, thereby inducing EMT in liver cancer cells (17). LINC01133 was found to be primarily located in the cytoplasm of cervical cancer cells, wherein it binds to miR-4784 and competitively inhibits the binding of AHDC1 mRNA to miR-4784. This results in the enhanced expression of AHDC1; thereby promoting the proliferation, migration, and EMT of cervical cancer cells (4). The oncogenic and tumor suppressor effect of LINC01133 *via* the ceRNA mechanism in various cancers have been presented in **Table 1**.

**Table 1:** The ceRNA mechanism of LINC01133 in cancers.

Types of Cancers		Binding miRNAs	Target mRNAs	Downstream Molecular Functions	Function of LINC01133	Cited Reference
Female Reproductive Cancers	Ovarian cancer	miR-205	LRRK2	Inhibition of Proliferation, Migration, and Invasion	Cancer suppressor	(27)
	Ovarian Cancer	miR-495-3p	TPD52	Enhancing Effect of Metastasis	Oncogene	(15)
	Cervical Cancer	miR-4784	AHDC1	Enhancing Effect of Proliferation and Migration Inhibition of Apoptosis	Oncogene	(4)
	Cervical Cancer	miR-30a-5p	FOXD1	Acceleration of proliferation and metastasis	Oncogene	(28)
Digestive Cancers	Gastric Cancer	miR-106a-3p	APC	Suppression of EMT and Metastasis	Cancer suppressor	(26)
	Gastric Cancer	miR-576-5p	SST	Suppression of Malignant Phenotypes	Cancer suppressor	(16)
	Pancreatic cancer	miR-216a-5p	TPT1	Suppression of Growth and Migration	Cancer suppressor	(29)
	Hepatocellular carcinoma	miR-199a-5p	Snail	Acceleration of Proliferation and Aggressive Phenotypes	Oncogene	(17)
Skeletal Cancer	Osteosarcoma	miR-422a		Aggravation of proliferation, migration, and invasion	Oncogene	(6)
urinary system cancer	Renal cancer	miR-30b-5p	Rab3D	Acceleration of Proliferation, Migration, and Invasion	Oncogene	(3)

### LINC01133 Binds to Proteins in the Cytoplasm

LINC01133 regulates protein function by directly binding to proteins to activate or inhibit its expression or by blocking its post-translational modification. Using an RNA pull-down assay and mass spectrometry analysis, Yi et al. showed that LINC01133 has nine potential binding proteins (with a peptide count > 5 and a unique peptide count > 5). The interaction between LINC01133 and ANXA2 had the highest score for protein identification analysis and was also identified with the RNA immunoprecipitation chip (RIP) assay in MHCC97H hepatocellular carcinoma (HCC) cells. ANXA2 was found to have higher expression levels in HCC tissues than that in matched peritumoral tissues, and this higher expression level of ANXA2 represented a shorter tumor-free survival period in the Kaplan-Meyer analysis. The phosphorylation levels of ANXA2 and STAT3 and the expression level of cyclin D1 were both increased by LINC01133 overexpression and decreased by its silencing. In conclusion, LINC01133 promotes HCC progression by binding to and promoting ANXA2 and STAT3 signaling (17). Similarly, Kong et al. overexpressed LINC01133 in HCT116 colon cancer cells and found that an increase in LINC01133 expression significantly repressed the migration and invasion of CRC cells. In addition, RNA pull-down and mas spectroscopy screening assays showed the direct binding of SRSF6 and LINC01133, which was confirmed using western blot and RIP assays. However, SRSF6 promoted CRC metastasis by inducing EMT independent of LINC01133, and the malignant phenotype was altered significantly by depleting SRSF6 in HT29 cells with high LINC01133 expression, in HCT116 cells with low LINC01133 expression, and in SW620 cells without LINC01133 expression. However, silencing LINC01133 in both SRSF6-expressing or -knocked down cells led to an interesting observation. The effect of LINC01133 silencing on EMT markers was attenuated in SRSF6-depleted cells, indicating that LINC01133 inhibited EMT and metastasis in CRC cells by binding to SRSF6 and blocking its function (17) (**Figure 2B**).

### LINC01133 Regulates Transcription by Binding to RNA-Binding Proteins (RBPs) in the Nucleus

LncRNAs localized in the nucleus regulate the transcriptional expression of multiple proximal or distal genes by binding to chromatin, transcription factors, or RBPs. Approximately 20% of lncRNAs have been reported to associate with polycomb repressive complex 2 (PRC2) and induce the trimethylation of H3K27 (30). Song et al. suggested that LINC01133 potentially suppresses SOX4 expression using this mechanism in breast cancer cells. RIP assay using an EZH2 antibody, which is an important subunit of the PRC2 complex, and RNA pull-down assay identified the interaction between EZH2 and LINC01133. Chromatin immunoprecipitation (ChIP) assay in LINC01133-overexpressing cells revealed that LINC01133 increased EZH2 and H3K27me3 at the promoter region of SOX4 to regulate its transcription and inhibit its expression (31). Weng et al. also found that LINC01133 binds to the promoter region of DKK1, resulting in H3K27 trimethylation and decreased DKK1 expression; while the expression of Wnt-5a, MMP-7, and β-catenin increased upon LINC01133 binding to their respective promoting regions in pancreatic cancer (32). Similarly, Zang et al. found that the nucleus-localized LINC01133 could interact with EZH2 and LSD1 and repress the transcription of KLF2, P21, or E-cadherin by recruiting EZH2 and LSD1 to the promoter regions of the inhibited genes in non-small cell lung cancer (33).

In nasopharyngeal carcinoma (NPC), RNA pull-down and RIP assays was used to validate the binding of YBX1 and LINC01133. YBX1 is a protein that can activate Snail translation and induce EMT. YBX1 knockdown inhibited the upregulation of cell migration and invasion *in vitro*, and this was affected by the depletion of LINC01133. This suggests that LINC01133 promoted NPC tumorigenesis by inhibiting YBX1 (8) (**Figure 2C**).

## Mechanisms Regulating the Expression Level of LINC01133

**Figure 3:**
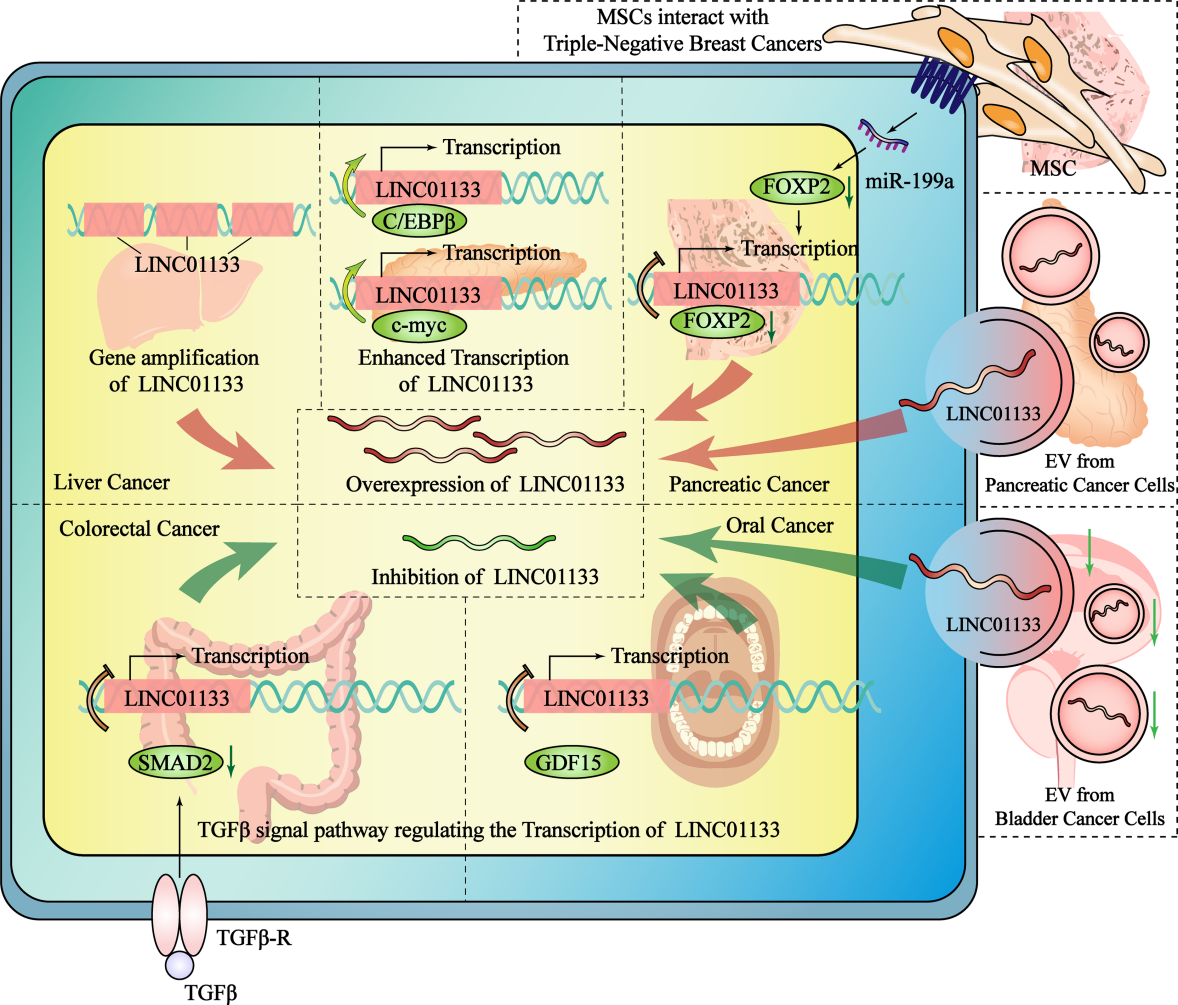
The mechanisms regulate the expression of LINC01133 in the cancers of different tissue type. The overexpression of LINC01133 could be regulated by gene amplification of LINC01133, the enhanced transcription of LINC01133, the increased transport of LINC01133 by EVs and the promoting effect from MSC in TME. The inhibition of LINC01133 could be regulated by the TGF-β pathway. GDF15 belongs to TGF-βsubfamily and also inhibit LINC01133 transcription. Compared with normal tissue cells, the number of LINC01133-containing exosomes secreted and received by cancer cells is reduced, which is one of the reasons why the concentration of LINC01133 in tumor cells decreases. The red arrow indicates the mechanisms that promote the expression of LINC01133, while the green arrow indicates the mechanisms that inhibit it. The black dashed lines separate different studies of the mechanisms that regulate the expression of LINC01133.

The abnormal expression of LINC01133 is extremely common in cancers. Therefore, elucidating the specific mechanism of regulating LINC01133 expression in tumors is of great significance for cancer therapies targeting the abnormal expression of LINC01133. Recently, studies have shown that the differential regulatory mechanism of LINC01133 primarily includes an increase in copy number and the regulation of transcription elements and entry from the tumor microenvironment (TME) (**Figure 3**). 

### Gene Amplification of LINC01133

Multiple duplications of the LINC01133 gene may directly increase its RNA expression. It has been reported that lncRNA gene amplification occurs frequently in liver cancer. Screening of copy number variations from the whole genome sequencing data of 49 Chinese patients and a validation cohort of another 238 patients with HCC revealed that the copy number and expression level of LINC01133 were significantly higher in HCC tissue than that in the para-cancer tissue. Additionally, there was a positive relationship between these two indices (R^2 =^ 0.535), indicating that a high amplification at the level of the DNA might be the cause of LINC01133 dysregulation (17).

### The Regulation of LINC01133 Transcription

Two studies on pancreatic ductal adenocarcinoma (PDAC) have revealed the underlying mechanism of transcription factor-mediated regulation of LINC01133 expression. Higher expression of LINC01133 and CCAAT/enhancer-binding protein β (C/EBPβ) was observed in PDAC compared to that in control conditions. C/EBPβ was found to positively regulate LINC01133 expression by binding to response elements within the LINC01133 promoter. The high expression of C/EBPβ was an indicator of a poor prognosis of PDAC (11). C-myc also binds to the promoter region of LINC01133 and is a key regulator of expression levels. C-myc overexpression was induced using periostin, which is a 90 kDa protein that is specifically secreted by pancreatic stellate cells, and this resulted in enhanced LINC01133 expression compared to that in control conditions (34). In oral squamous cell carcinoma, a transcriptional regulatory mechanism was found to regulate LINC01133 expression. The TGF-β canonical signaling pathway-mediated regulation of transcription is known to be associated with the occurrence and development of malignant tumors (35). The transcription of LINC01133 is also regulated by the TGF-β superfamily. In CRC, TGF-β inhibits the transcription of LINC01133, leading to EMT (36). In a study by Kong et al., a negative feedback inhibitory pathway was found between the TGFβ superfamily member GDF15 and LINC01133, causing a decrease in LINC01133 expression in oral squamous cell carcinoma and thereby promoting tumor metastasis (9).

### The Regulation of Entry of LINC01133 in Extracellular Vesicles Secreted by Cells in the TME

In addition to endogenous LINC01133 present in cancer cells, exogenous LINC01133 present in exosomes in the TME can also affect the malignancy of cancer cells. Extracellular vesicles (EVs) or exosomes may originate from constituent cells in the TME, even from tumor cells themselves. Liu et al. have reported that the high secretion of exosomes containing LINC01133 in PDAC is correlated with a poor overall survival rate of PDAC patients. In their study, periostin was found to promote exosome secretion and to induce EMT. They demonstrated that exosomal LINC01133 extracted from a LINC01133-overexpressing cell line promoted proliferation, migration, invasion, and EMT and inhibited apoptosis in PDAC cells. In order to identify the mechanism underlying the regulation of LINC01133 expression, the nuclear localization of LINC01133 was first confirmed by a fluorescence *in situ* hybridization assay. Then, chromatin isolation by RNA purification and CHIP assay was used to identify a binding interaction between EZH2 and LINC01133 that promotes H3K27 trimethylation and inhibits the transcription of AXIN2 by targeting its promoter region. LINC01133-mediated silencing of AXIN2 further suppressed GSK3 activity, ultimately activating β-catenin (34). In bladder cancer (BC), LINC01133 expression was found to be high in exosomes of the SV‐HUC‐1 human uroepithelial cell line compared to the low expression in BC cells. Similar to the function of LINC01133, exosomes containing LINC01133 inhibited cell viability, proliferation, migration, and invasion. Exosomal LINC01133 was found to repress BC progression by regulating the Wnt signaling pathway (7).

### Function of LINC01133 in Cancers

**Figure 4:**
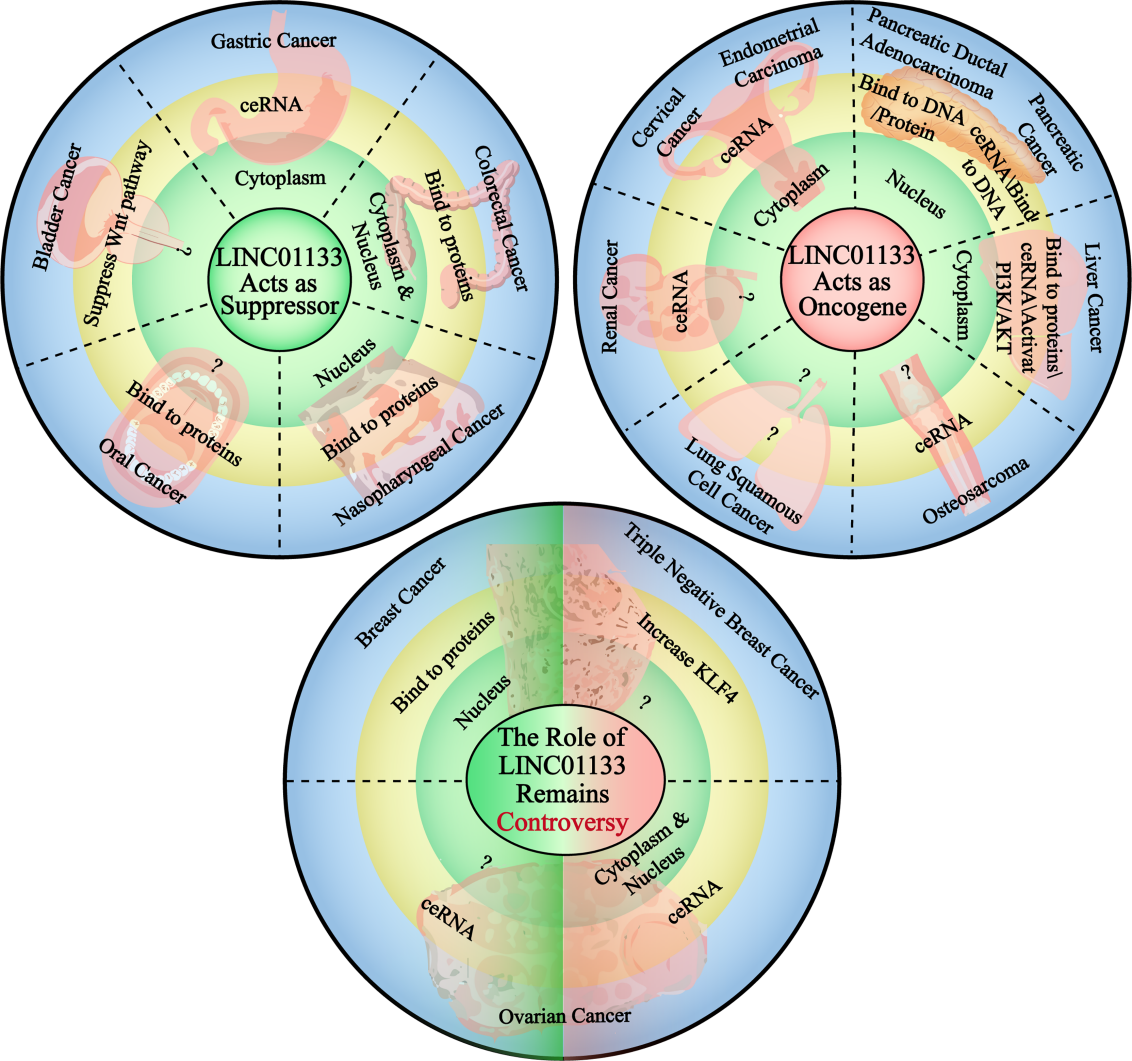
The carcinogenetic or suppressing role of LINC01133 in the cancers of different tissue type. The circle in the upper left corner demonstrates the cancers in which LINC01133 acts as a cancer suppressor, the subcellular localization of LINC01133 in each type of cancer and the main mechanism of it to function. The circle in the upper right corner demonstrates the cancers in which LINC01133 acts as an oncogene, and also the subcellular localization and functional mechanisms. The circle lower in the middle states the cancers in which the role of LINC01133 is still controversy. The green left side represents the suppressor role of LINC01133, the localization and functional mechanisms, and the red right side represents the oncogene role of LINC01133, the localization and functional mechanisms. The black dashed lines separate the cancers of different tissue types.

The complex and diverse molecular regulatory mechanisms of LINC01133 determine the molecular basis for its functional specificity in different cancers. The abnormal expression and dysfunction of LINC01133 in different cancers are distinct in nature (**Figure 4**).

### LINC01133 Functions as a Cancer Suppressor

LINC01133 functions as a tumor suppressor in CRC. Compared with 219 normal tissue samples, LINC01133 was found to be significantly downregulated in CRC tissues. The low expression of LINC01133 was positively correlated with tumor metastasis, TNM staging, and low overall survival rate of CRC patients. Attenuating LINC01133 function by shRNA or miRNA binding promotes metastasis of CRC cells both *in vivo* and *in vitro (* 36). In gastric cancer, the low expression and anti-tumor effect of LINC01133 was predicted using bioinformatics analysis and verified experimentally as well. Yang et al. quantified the level of LINC01133 in 200 cases of gastric cancer and paired adjacent tissues using qPCR and found a significantly lower expression level of LINC01133 in gastric cancer tissues compared to that in the paired tissues (26). This result was also consistent with that of another study that analyzed a cohort of 254 pairs of gastric cancer and adjacent tissues (16). In both these studies, LINC01133 acted as a tumor suppressor *via* the ceRNA mechanism. Bioinformatics analysis using the Gene Expression Omnibus and The Cancer Genome Atlas databases and subsequent verification revealed a tissue-specific downregulation of LINC01133 expression in gastrointestinal tissues compared to that in control tissues (37). In **Table 2**, we summarize the types of cancers in which LINC01133 exerts a tumor suppressor effect and the underlying mechanism of action.

**Table 2:** Characterization of LINC01133 in cancers.

	Types of Cancers		Function of LINC01133	Subcellular localization	Mechanism	Cited Reference
Cancer Suppressor	Digestive Cancers	Gastric Cancer	Cancer suppressor	cytoplasm	CeRNA	(26)
		Gastric Cancer	Cancer suppressor	cytoplasm	CeRNA	(16)
		colorectal cancer	Cancer suppressor	cytoplasm and nucleus	Bind to proteins	(36)
		colorectal cancer	Cancer suppressor			(38)
		oral squamous cell carcinoma	Cancer suppressor		Bind to proteins	(9)
	Respiratory cancer	nasopharyngeal carcinoma	Cancer suppressor	nucleus	Bind to RBPs	(8)
	Urinary system cancer	bladder cancer	Cancer suppressor	exosomes	Suppress Wnt pathway	(7)
	Female Reproductive Cancers	Cervical Cancer	oncogene	cytoplasm	CeRNA	(4)
		Cervical Cancer	oncogene		CeRNA	(28)
		endometrial carcinoma	oncogene			(39)
Oncogene	Digestive Cancers	pancreatic ductal adenocarcinoma	oncogene	nucleus	Bind to DNA	(34)
		pancreatic ductal adenocarcinoma	oncogene		Bind to proteins	(11)
		Pancreatic cancer	oncogene	primarily in the nucleus	Bind to DNA	(32)
		Pancreatic cancer	oncogene		CeRNA	(29)
		hepatocellular carcinoma	oncogene	cytoplasm	CeRNA, Bind to preins	(17)
		Hepatocellular carcinoma	oncogene		Activate PI3K/AKT pathway	(40)
Controversy	Urinary system cancer	Renal cell carcinoma	oncogene		CeRNA	(3)
	Respiratory cancer	lung squamous cell cancer	oncogene			(41)
	Others	Osteosarcoma	oncogene		CeRNA	(6)
	Female Reproductive Cancers	breast cancer	Cancer suppressor	nucleus	Bind to RBPs	(31)
		Triple Negative Breast Cancer	oncogene		KLF4↑	(42)
		Ovarian Cancer	Cancer suppressor		CeRNA	(27)
			oncogene	cytoplasm and nucleus	CeRNA	(15)

### LINC01133 Functions as an Oncogene

In most gynecological cancers, LINC01133 plays the role of an oncogene. Yang et al. found that LINC01133 is highly expressed in endometrial cancer and its high expression was positively correlated with the occurrence of this cancer. Knockdown of LINC01133 reduced the vitality of endometrial cancer cells by arresting the cell cycle and increasing apoptosis of cancer cells, while simultaneously inhibiting their migration and invasion ability *in vitro (* 39). LINC01133 levels were also found to be significantly elevated in the serum of patients with cervical squamous carcinoma (CESC) and cervical intraepithelial neoplasia (CIN) when compared to that in the control group; but there was no difference in LINC01133 expression between the CESC and CIN groups (43). Moreover, high LINC01133 levels were associated with a shorter survival period in CSEC patients (44). LINC01133 is also likely to function as an oncogene in non-digestive tract cancers. An *in vitro* study by Zheng et al. showed that the expression of LINC01133 was increased in liver cancer cell lines compared to that in control cells, and this increased expression promoted malignancy in cancer cells *via* the PI3K/AKT pathway (40). This was consistent with the result of Dan Yin’s study. Further, a ceRNA network between miR-199a-5p, LINC01133, and SNAI1 was identified to regulate the growth, invasion, and metastasis of liver cancer cells (17). Several studies have reported the carcinogenicity of LINC01133 in pancreatic cancer, (**Table 2**). In urinary system cancers, LINC01133 has been found to act as an oncogene in renal cancer cells. Zhai et al. reported that LINC01133 was highly expressed in renal cancer cell lines and in 34 renal cancer tissue specimens when compared with that of normal cell lines and tissues. The depletion of LINC01133 inhibited the proliferation, migration, and invasion of renal cancer cell lines. *In vivo* knockdown of LINC01133 inhibited subcutaneous tumor formation in nude mice (3). Details of the oncogenic function of LINC01133 in cancer are shown in **Table 2**.

### Contradictions in LINC01133 Function

The role of LINC01133 in some gynecological tumors remains controversial. For example, Song et al. found that LINC01133 expression was significantly downregulated in breast cancer samples compared to that it control samples, and this was associated with the progression and poor prognosis of breast cancer. As mentioned previously, the overexpression of LINC01133 inhibits the invasion and metastasis of breast cancer cells both *in vitro* and *in vivo via* a transcription-mediated regulatory mechanism (31). However, a study by Zhenbo Tu showed that mesenchymal stem/stromal cells (MSCs) played a critical role in promoting the initiation and progression of triple-negative breast cancers by inducing LINC01133 expression. In this study, LINC01133 promoted the phenotypic and growth characteristics of cancer stem cell-like cells and was a direct mediator of the MSC-triggered miR-199a-FOXP2 pathway (42). A similar contradiction regarding LINC01133 function also exists in ovarian cancer. Liu et al. (2019) found that LINC01133 expression was reduced significantly in ovarian cancer when compared with that in normal tissue, and this downregulation was an indicator of poor prognosis. The low levels of LINC01133 regulated by miR-205 promoted tumor proliferation and metastasis both *in vivo* and *in vitro* (27). However, in a study by Liu et al. (2020), LINC01133 was reported to be upregulated in epithelial ovarian cancer tissues and cell lines compared with that in control tissues and cell lines, and this increase facilitated cancer cell migration and invasion *in vitro* and tumor metastasis *in vivo* (15). Detailed information regarding the contradictory functions of LINC01133 had been presented in **Table 2**.

## LINC01133 as a Potential Biomarker and Therapeutic Target in Human Cancer

**Figure 5:**
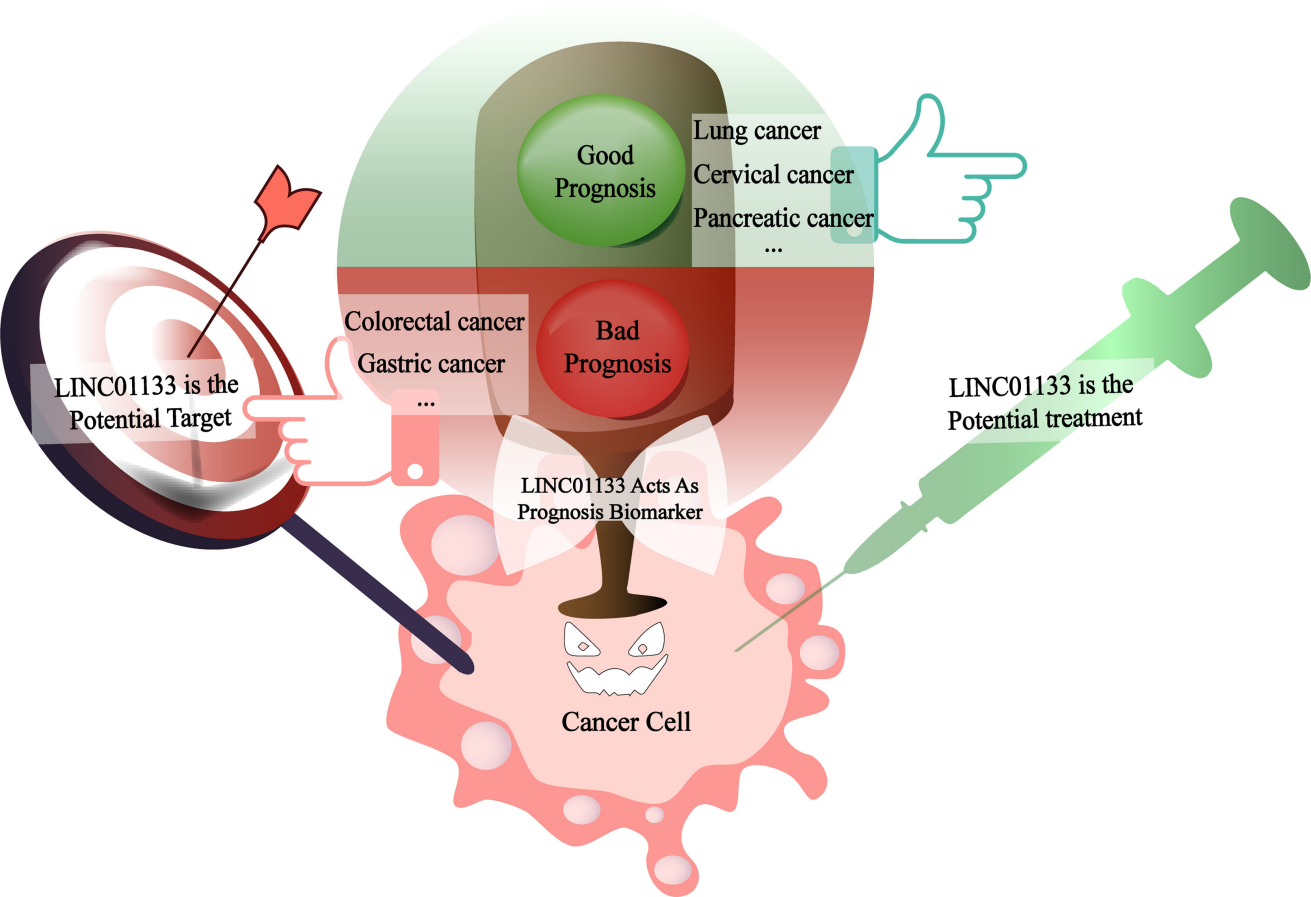
LINC01133 is the biomarker of cancer prognosis as well as the potential therapeutic target or treatment reagent in the future. When LINC01133 acts as an oncogene, its high expression suggests a bad prognosis of patients as shown in the red section and might be a potential therapeutic target molecular for the clinical treatment. In the contrary, when LINC01133 acts as a cancer suppressor, its high expression indicates a good prognosis of patients as shown in the green section and might be a potential treatment reagent in the future.

The abnormal expression of LINC01133 in cancers makes it a potential biomarker of the disease. In some cancers with well-defined and -documented effects, LINC01133 has great potential to be a prognostic marker. For example, the results of bioinformatics analysis showed evidence of a correlation between high LINC01133 expression and poor PDAC prognosis. LINC01133 was identified by weighted gene co-expression network analysis, and a survival analysis was performed to verify its role in advancing cancer progression (45). In pancreatic cancer (45), cervical cancer (44), and lung adenocarcinoma (5), increased LINC01133 levels represent poor patient survival. While in CRC (38), gastric cancer (26), and other cancers, a high level of LINC01133 was an indicator of a good prognosis (**Figure 5**).

As mentioned, the abnormal expression of LINC01133 directly affects the prognosis of cancer patients. The molecular mechanisms underlying its biological functions are gradually being elucidated. Therefore, in cancers where LINC01133 exerts a carcinogenic effect, LINC01133 is a potential target for cancer therapies. However, considering that LINC01133 acts as a tumor suppressor gene in some cancers, importing functional LINC01133 is also a viable treatment option. Recent studies have shown that LINC01133 can be encapsulated into EVs (7, 34) and transported into tumor cells to regulate the traits of cancer cells. With further advances in research that aims to develop EVs as targeted drug carriers, it will become possible to deliver LINC01133 as a targeted therapeutic drug to cancer foci *via* EVs in the future (**Figure 5**).

## Future Directions for LINC01133 Research

Although several diverse studies have examined the regulatory mechanism of LINC01133 on cancer; as a non-coding RNA with obvious tissue-specific expression, further research in this area is still needed. Various mechanisms that can affect the function and expression of lncRNA; such as mutation (46), amplification or deletion of lncRNA at the genomic level (20), DNA methylation (47), histone modification (48), RNA modification (for example, m6A) (49), binding with RBPs [for example, transcription factor (50) or transcription enhancer (51)], and the interaction with bound RNAs (for example, miRNA) at the epigenetic level are all potential future directions in understanding the regulation of LINC01133 expression.

Further, additional mechanisms underlying the biological function of LINC01133 still need to be explored. Considering that lncRNAs require proteins to execute their regulatory roles, proteins that directly or indirectly interact with LINC01133 need to be identified. RBPs are well known for their roles in regulating RNA fate (from synthesis to decay) and protein translation (by assisting and/or directing RNAs) (52). However, the only RBPs reported to be associated to LINC01133 are the translation-promoting protein YBX1 (8) and the transcription repressor EZH2 (31). Therefore, cross-linking immunoprecipitation and RIP assays can be used to identify RBPs that bind to LINC01133.

More notably, the impact of the TME on cancer cells is receiving increasing attention (53–55), and the transport and protection of lncRNAs by exosomes when secreted in the TME to regulate malignancy of cancers is also a new mechanism that has been recently discovered (56–58). Numerous studies have found that lncRNAs can be secreted from different types of TME stromal cells, including cancer-associated fibroblasts (57), cancer-associated macrophages (59), and cancer cells packaged in EVs. EVs act as carriers of lncRNAs and are swallowed by cancer cells to allow functional lncRNAs to enter tumor cells and execute their biological functions. LINC01133 has been shown to be secreted into EVs from pancreatic ductal adenocarcinoma cells and is transferred to cancer cells to silence AXIN2/GSK3 and activate the Wnt/β-catenin pathway, which promotes EMT (34). In bladder cancer, LINC01133 is packaged into EVs from both cancer and normal tissue cells. Interestingly, the concentration of LINC01133 in EVs from normal tissue cells was higher than that in EVs from cancer cells. EVs are taken up by cancer cells, wherein they regulate the Wnt signaling pathway and inhibit the progression of bladder cancer (7). However, the types of stromal cells that secrete EVs containing LINC01133, the function of LINC01133 in the stromal cell itself, and the distinct roles of LINC01133 in the TME of different tissues are yet to be elucidated.

## Conclusion

Similar to other star lncRNA molecules, the functions and underlying mechanisms of LINC01133 in cancers of different tissue types needs to be extensively studied. In future studies, additional mechanisms affecting the expression and regulatory mechanisms of LINC01133 should be explored. In addition, the role of LINC01133 in the interaction between cells in the TME should be studied in different cell types and in EVs. Due to the current contradiction with respect to its carcinogenic and tumor suppressor status, LINC01133 is a potential target for cancer therapies and a candidate molecule for the development of tumor treatment reagents.

## Author Contributions

SJ: Conceptualization, Investigation, Writing – original draft, Writing – review &editing. QZ: Writing-original draft, Writing – review &editing. JL: Writing – review &editing. KR: Language editing. XK, SL, and CS: Visualization, review & editing. XL: Writing – original draft. DZ: Conceptualization, review & editing. SF: Conceptualization, editing& Funding acquisition. MC: Conceptualization, Investigation, Writing – original draft, Writing-review, editing & Funding acquisition. All authors contributed to the article and approved the submitted version.

## Funding

The present study was supported by HMU Marshal Initiative Funding (No. HMUMIF-21007) and the Open Project Program of Key Laboratory of Preservation of Human Genetic Resources and Disease Control in China (Harbin Medical University), Ministry of Education (No. LPHGRDC2020-001).

## Conflict of Interest

The authors declare that the research was conducted in the absence of any commercial or financial relationships that could be construed as a potential conflict of interest.

## Publisher’s Note

All claims expressed in this article are solely those of the authors and do not necessarily represent those of their affiliated organizations, or those of the publisher, the editors and the reviewers. Any product that may be evaluated in this article, or claim that may be made by its manufacturer, is not guaranteed or endorsed by the publisher.

## Abbreviations

CeRNA, competing endogenous RNA; CRC colorectal cancer,EMT, epithelial-mesenchymal transition; EVs, extracellular vesicles; HCC, hepatocellular carcinoma; LncRNA, long non-coding RNA; LincRNA, long intergenic non-coding RNA; LINC01133, long intergenic non-coding RNA01133; PDAC, pancreatic ductal adenocarcinoma; RBPs, RNA binding proteins; RIP, RNA immunoprecipitation chip assay; TME, Tumor micro-environment.
